# A pilot randomized controlled trial examining the feasibility of perioperative rehabilitation for inguinal hernia repair surgery

**DOI:** 10.1371/journal.pone.0324907

**Published:** 2025-05-22

**Authors:** Anna Shologan, Omar Farooq, Geoffrey Bostick, Luciana Macedo, Quentin Durand-Moreau, Meaghan Ray Peters, Douglas P. Gross

**Affiliations:** 1 Faculty of Rehabilitation Medicine, University of Alberta, Edmonton, Alberta, Canada; 2 Department of Surgery, University of Alberta, Edmonton, Alberta, Canada; 3 School of Rehabilitation Science, Faculty of Health Sciences, McMaster University, Hamilton, Ontario, Canada; 4 Division of Preventive Medicine, University of Alberta, Edmonton, Alberta, Canada; Faculty of Medicine of Monastir, TUNISIA

## Abstract

**Background:**

Despite the high frequency of inguinal hernia repair (IHR) surgery, there is little research investigating pre- or post-operative exercise and education in this population. Recommendations regarding perioperative physical activity are inconsistent and largely based on clinical opinion. We conducted a pilot randomized controlled trial to examine the feasibility of perioperative rehabilitation for inguinal hernia repair surgery in terms of recruitment rate, assessment, and protocol implementation.

**Methods:**

Participants were randomized into an intervention group and control group. Descriptive and patient-reported data were collected through online surveys at baseline, post-prehabilitation (prehab), 1-week post-operative (post-op), and 12-week post-op. Eligible participants completed a performance-based modified Short Form Functional Capacity Evaluation conducted by a masked observer at baseline, after 6 weeks of exercise and/or education, and at 12-week post-op. Participants in the intervention group received 6 weeks of exercise and education prior to and then after surgery. The control group received care as usual.

**Results:**

Thirty-one participants awaiting IHR with a mean age of 49 years were recruited (recruitment rate of 51.7%). Thirty participants were randomized into control (n = 16) and intervention groups (n = 14), while 1 dropped out prior to beginning the study due to being unable to take time off work for assessment. Twenty-four participants completed the final 12-week post-op follow-ups. Twenty-one participants returned to work by the 12-week post-op follow-up. Sixty-seven percent of participants in the intervention group exercised at least 3 times per week post-operatively. One participant in each group experienced exacerbations of hernia symptoms that were unrelated to study activities. Functional testing resulted in minimal symptom exacerbation in either group, but the intervention group reported less pain at 12-week post-op than controls.

**Conclusion:**

A randomized trial of perioperative rehabilitation for patients undergoing inguinal hernia repair appears feasible, but protocol adjustments are needed to improve recruitment rate, assessment, and participant retention.

**Trial Registration:** This trial is registered with ClinicalTrials.gov Identifier: NCT05069142

## Background

Inguinal hernia repair (IHR) surgery is one of the most common surgeries performed around the globe, with an estimated 20 million procedures annually [1]. Despite the frequent occurrence of this condition and consequent surgical repair, patient outcomes for IHR need improvement; the rate of hernia recurrence following surgical repair is approximately 15% and an estimated 10–12% of patients undergoing this surgery have chronic post-surgical pain that lasts months or years [2]. Poor surgical outcomes can negatively impact an individual’s life leading to significant activity limitation and decreased quality of life. Because IHR surgery is so common, even modest improvements in clinical outcomes could have a significant impact for many people [3].

There is a lack of quality literature investigating the effects and safety of perioperative care, including physical activity, surrounding inguinal hernia. Consequently, current recommendations given to patients are variable, inconsistent, and widely based on clinical opinion. While technologies and techniques for the procedure continue to evolve and improve, there is limited research investigating whether better surgical preparation through pre-operative exercise and education (i.e., prehabilitation) followed by post-surgical rehabilitation results in improved outcomes following IHR surgery. Evidence from related procedures indicates that better surgical preparation through structured pre-operative exercise and standardized education followed by ongoing post-surgical rehabilitation leads to more rapid recovery, return to activity, and lower likelihood of persistent post-surgical pain [4,5]. Current evidence suggests that a general increase in physical capacity and resilience likely improves outcomes during and following abdominal surgeries [4–9]. The combination of prehabilitation and post-operative rehabilitation is termed perioperative rehabilitation.

Specific to IHR, Liang et al. [10] was the first to conduct a randomized controlled trial (RCT) examining the effects of prehabilitation in 118 patients undergoing ventral hernia repair. While they found benefits to prehabilitation, it was also associated with higher risks such as a higher rate of dropout and need for emergent repair. The primary presurgical goal for the intervention group in this study was weight loss and the length of prehabilitation was non-standardized making protocol recommendations difficult to extrapolate. The most relevant literature pertaining to IHR rehabilitation is a dated case study that used an occupational rehabilitation approach following surgical repair and saw an airline baggage attendant return to regular activity far sooner than typical post-operative recommendations [11,12]. Related studies have examined how the concepts of prehabilitation and rehabilitation can be applied to abdominal surgeries including ventral hernia repair [13–15]. These studies all suggest that improved physical stamina results in improved surgical outcomes, however little description is provided regarding what type of interventions were examined.

Despite the relative simplicity and high frequency of inguinal hernia repair (IHR) surgery, there is little research investigating pre- or post-operative exercise and education in this population. We examined the feasibility of clinical trial methods for evaluating perioperative rehabilitation for inguinal hernia repair surgery in terms of recruitment rate, assessment, and protocol implementation.

## Methods

### Design

We conducted a two-armed pilot randomized controlled trial (RCT, Identifier: NCT05069142). This included the development and testing of a structured exercise and education program, tailored to individual patient’s needs, and conducted before and after the IHR surgery to reduce the likelihood of chronic post-surgical pain and increase functional abilities while doing minimal harm. Participants were randomized 1:1 to intervention and control groups using an allocation sequence generated within the Research Electronic Data Capture (REDCap) program by a study team member (LM) not involved in randomization.

Ethics approval was obtained through the University of Alberta’s Health Research Ethics Board (Study ID: Pro00106451). Written participant consent was obtained online via REDCap.

### Sample

Between February 8, 2022 and October 12, 2022 we enrolled patients referred to general surgery for elective IHR within one Alberta Health Services (the provincial and public integrated healthcare system) general surgery clinic. We enrolled patients undergoing first-time IHR surgery. Patients were invited to participate in our study by a surgeon on our study team (OF) after undergoing a thorough medical examination; all patients received a similar open surgical technique with the same surgeon (OF).

Inclusion criteria included:

1) Scheduled to undergo first-time IHR surgery after a physical examination by a surgeon identified signs and symptoms consistent with inguinal hernia (direct or indirect hernia).
2) Willingness to participate in a 6-week targeted exercise program.
3) At least 18 years of age.
4) No medical contraindications to participation in exercise: this included uncontrolled medical conditions such as diabetes, hypertension, vertigo, congestive heart failure, chronic obstructive pulmonary disease, intra-abdominal ascites, or pre-existing malnutrition.
5) Full-time workers reporting they had to lift at least 10 kg (22lb) occasionally (3–33% of the workday) at work.

Exclusion criteria included:

1) Recurrent hernia.
2) Body Mass Index >35.0 kg/m² since morbidly obese patients experience more surgical complications [15].
3) Use of narcotics.
4) Poorly controlled bone and joint conditions of the spine or extremities.
5) History of other abdominal surgeries that have resulted in a permanent lifting restriction.

### Sample size

Since this is a pilot study evaluating feasibility of our intervention and evaluation protocols, we aimed to enrol 30 patients and randomize 15 to each treatment group. This sample size has been found to provide adequate information to determine feasibility and whether minor or major modifications are advisable [16].

### Data collection procedures

Participation was voluntary, and patients were invited to participate in the study at least 8 weeks before surgery. Baseline testing included a clinical assessment done by a physical therapist along with a performance-based functional assessment (described in detail below) [17].

Following baseline assessment, participants in the intervention group then received perioperative exercise instruction from a clinical exercise physiologist (AS). Participants in both groups received educational videos on pain self-management and expectations surrounding the surgical and recovery process. Participants in the intervention group received additional education regarding pre- and post-operative exercise guidelines. Three weeks after surgery, participants in the intervention group gradually progressed through post-operative exercises and modifications were made as needed. The exercises avoided all contraindications in the acute post-surgical period, including no lifting >10 kg for the first 4 weeks. The study did not impact the scheduling of surgery or typical procedures. See Appendix A for activities and timing of data collection as outlined in “Perioperative Rehabilitation Activity and Data Collection Protocol for Inguinal Hernia”.

### COVID-19 implications

As this study was designed and conducted during the global COVID-19 pandemic, this had implications for project planning and execution. Since IHR operations continued to be conducted in Alberta despite the pandemic and physiotherapy clinics were following public health recommendations, this study was conducted within the typical clinical care pathway. In-person interactions were kept to a minimum and all interventions were conducted in accordance with COVID-19 requirements in place at the time.

### Blinding

To minimize observer bias, the physical therapist conducting performance-based functional testing was blinded to which group participants were randomized into. Participants were informed which treatment group they were randomized into after baseline functional testing was completed. Blinding participants to which treatment group they were in was not possible since the primary intervention was structured exercise.

### Intervention description

The perioperative rehabilitation protocol was developed by our research team after a literature review. The protocol included an established set of structured exercises aimed at core strengthening in pre- and post-operative stages. We followed a traditional occupational rehabilitation approach, focusing exercise training on activities that the patient was expected to have trouble with due to the hernia. When providing exercise instruction, emphasis was placed on proper breathing techniques while performing exercises to avoid the Valsalva maneuver and avoid an increase in intrathoracic pressure [18]. Goals for prehabilitation were primarily improving general neuromuscular control as morphological changes are not typically seen within a six-week period [19]. Neuromuscular adaptations within this early training period often result in improved physical performance due to changes in coordination and improved muscle recruitment and activation during specific tasks [19]. The program was delivered in-person by a qualified exercise professional using the commercially available web-based Physitrack exercise programming application (ca.physitrack.com) and follow-ups were conducted virtually.

The exercise protocol was developed with consideration for what exercises would be safe for this patient population, provide maximum benefit for their condition, simple to implement and easy for patients to learn. Exercises chosen included diaphragmatic breathing, abdominal bracing (transverse abdominis activation), bridging, dead-bugs, bird-dogs, push-ups, chair squats, and floor-to-waist lifting. See Appendix B: “Perioperative Rehabilitation Protocol for Inguinal Hernia Repair” for a detailed outline of exercise protocol implementation. These exercises were all easily modifiable to make them more or less challenging for individual participants as needed. Exercise frequency and reported pain levels were recorded weekly for six weeks following baseline assessment and initial instruction, and then post-operatively beginning 3 weeks after surgery. Post-operative exercises were gradually reintroduced, starting with simpler exercises and progressing weekly in difficulty and intensity.

Education was provided in-person by the surgeon and the study team, as well as through YouTube videos that provided standardized information. Educational videos were created by study team members based on available best practice guidelines related to IHR surgery as well as pain education. Participants were encouraged to watch the videos on their own and were sent the web links at baseline and one week post-operatively. Education provided included basic information about inguinal hernia and the surgical repair technique, what to expect in the days and weeks following surgery, advice for post-operative recovery, the importance of activity and exercise to recovery, pain coping strategies and techniques for reducing the risk of hernia recurrence. See Appendix C for web links to educational videos.

## Measures

### Performance-based functional assessment

In-person performance-based testing was completed at baseline, following six weeks of exercise and/or education program, and 12-weeks post-surgical IHR by a researcher (MRP) not involved in the delivery of the intervention. Assessment protocol included a standardized Short Form Functional Capacity Evaluation (SF-FCE) shown to provide useful information for predicting return-to-work in people with trunk-related conditions [17,20]. Test items included 15-minutes of standing, maximum floor-to-waist lifting, 1-minute of crouching, 2-minutes of sustained forward flexion, and 5-minutes of repetitive trunk rotation [21]. Floor to waist lifting performance was stopped at 45 kg, representing very hard work. Throughout the performance testing participants wore a heart rate monitor to ensure heart rate remained below 80% of the estimated Heart Rate max. Biomechanical performance was also monitored for safety and participants were advised to terminate tasks if their hernia pain rating exceeded a 6/10.

An abdominal endurance test (horizontal plank test) and 30-second sit-to-stand test were added to the SF-FCE protocol to perform while stressing the hernia site during abdominal muscle activation and repetitive standing. The abdominal endurance test is a reliable tool to evaluate abdominal muscle fatigue [22], and the 30-second sit-to-stand test is commonly used by rehabilitation professionals as a valid indicator of lower extremity strength [23].

### Self-reported measures

Self-reported data were collected at baseline, 6–8 weeks after baseline at “post-prehab”, 1-week post-operatively, and 12-weeks post-operatively. Self-reported outcome measures and demographics were collected via online surveys through REDCap. Baseline surveys included demographic and health-related questions including age, gender, ethnicity, BMI, exercise frequency, smoking status, and job demands. Regular exercise was defined as ≥ 150 minutes per week of moderate to vigorous physical activity as outlined by the Canadian Society for Exercise Physiology guidelines [24].

Participants also completed clinical outcome measures including work status questionnaires, Numerical Pain Scales, the Pain Disability Index, and the Short Form-12 (SF-12) Health Survey. Questionnaires such as the Pain Disability Index and SF-12 Health Survey are easy to administer and comprehend, while providing reliable data before and after surgery [25,26]. The Carolinas Comfort Scale was completed post-operatively to measure symptoms related directly to IHR [27]. The Carolinas Comfort Scale is a self-report questionnaire with a maximum score of 115-points, where higher scores indicate worse symptoms. See Appendix A: Perioperative Rehabilitation Activity and Data Collection Protocol for Inguinal Hernia Repair.

### Feasibility measures

In addition to performance-based and self-report clinical information, we collected study protocol feasibility outcomes. Primary feasibility outcomes purpose-built by the research team included:

1) Recruitment percentage expressed as the number of participants enrolled in the program divided by the number of patients referred to the study.
2) Acceptability and compliance with study questionnaires.
3) Acceptability of functional testing protocol.

Full feasibility and acceptability criteria outlined using the traffic light system are described in detail below in Results [16].

### Safety and adverse effects

Since the prehabilitation program involves physical exercise by the participants, there was an inherent element of physical risk. This was explained to participants to possibly involve fatigue, tiredness, physical stress or injury, or other related complications. The prehabilitation program was in addition to each individual’s usual activity. With exercise, possible complications could arise before the operation is performed or after surgery. Prior to surgery, the patient could experience more pain in the hernia site as well as normal pain associated with exercise, they may notice worsening of the size of the hernia, and there is a very small chance of hernia incarceration.

At the virtual weekly sessions for participants in the intervention group, participants were asked about both exercise completion as well as any symptoms or adverse events experienced; these were documented. Increased symptoms were monitored, and exercises modified if the symptoms became intolerable. Severe complications (i.e., new severe pain, unexplained symptoms, etc.) were referred to the surgeon and any complications severe enough to warrant referral to the surgeon or ultrasound imaging were tracked.

### Statistical analysis

Descriptive statistics (mean, standard deviations, absolute and relative frequencies) were calculated to summarize the characteristics of our sample using analysis tools in REDCap and Microsoft Excel. Descriptive statistics were also calculated for functional testing, self-reported outcome measures, and feasibility outcomes at each time point and reported as mean. No statistical testing was completed as this was a pilot study of feasibility only.

## Results

### Feasibility results

**Fig 1:**
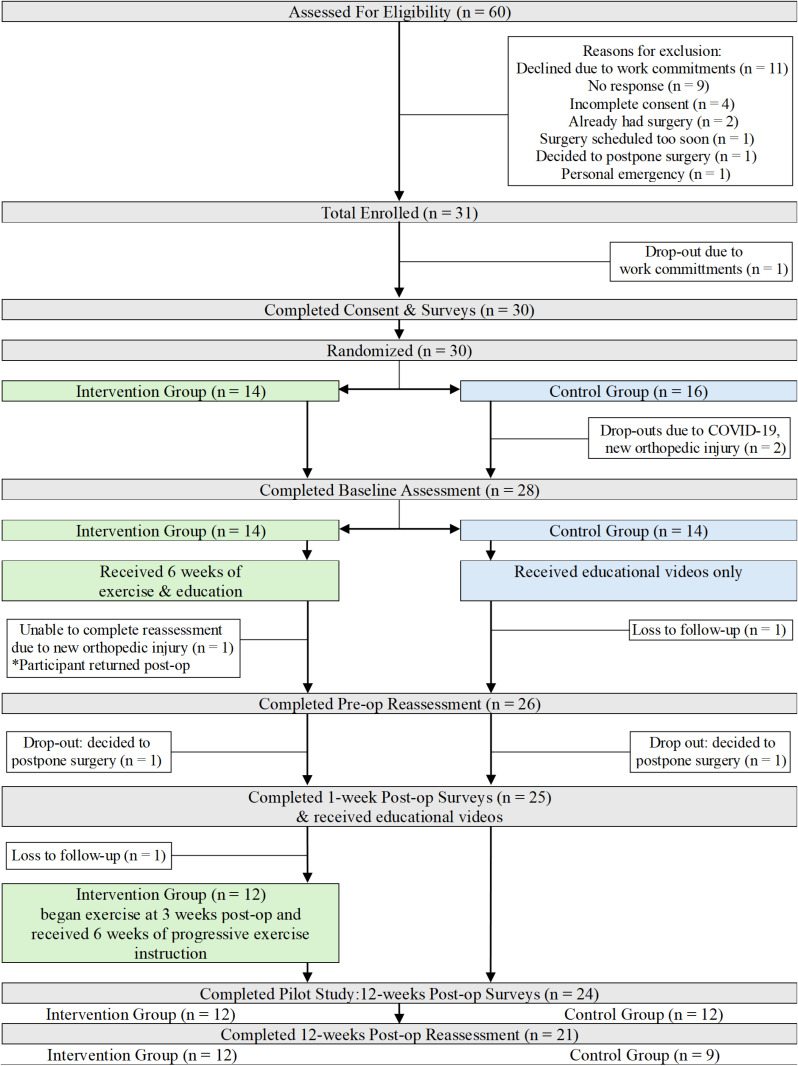
CONSORT flowchart illustrating participant recruitment.

Consolidated Standards of Reporting Trials (CONSORT) guidelines were followed as applicable for randomized pilot and feasibility trials [28]. See Fig 1 for a CONSORT flowchart illustrating participant recruitment and flow through the study.

Inclusion criteria were expanded to allow for 4 referrals who were not necessarily working full time (seasonally employed, working part time, or currently seeking employment), but were otherwise active, healthy, and willing to participate in the study. Exclusion criteria also required modification during the recruitment process, as during the early phases of the study the participating surgeon pointed out that excluding individuals who had any previous abdominal surgeries would lead to a significant decrease in number of referrals. We then screened referrals for any previous surgeries that resulted in a permanent lifting restriction.

Sixty patients referred to undergo elective IHR surgery were assessed for eligibility by the research team between February and October 2022. Of these, 31 were enrolled (recruitment rate: 51.7%). Reasons for non-enrollment included scheduling conflicts due to work or inability to take time off to attend baseline assessment (18.3%), no response (15.0%), incomplete consent to contact forms (6.7%), having already had IHR surgery (3.3%), were having the surgery too soon precluding participation in the intervention (1.7%), were having the surgery too far in the future (1.7%), and personal emergency (1.7%).

Of the 31 participants who agreed to participate, 30 provided informed consent and completed baseline surveys and 28 completed functional testing. One participant dropped out of the study after consenting due to work commitments prior to completing baseline surveys. In total 14 participants (46.7%) were assigned to the intervention group and 16 (53.3%) to the control group. The 2 participants who completed baseline surveys but were not able to complete in-person testing (1 due to acute COVID-19 infection and 1 due to new musculoskeletal injury) had both been assigned to the control group. Twenty-six participants completed in-person functional testing 6 weeks post-intervention. One participant in the intervention group was unable to complete the pre-op reassessment due to new musculoskeletal injury but re-entered the study following IHR surgery. Twenty-four participants completed the study to the final 12-weeks post-op surveys resulting in a retention rate of 77.4%.

Most participants (58%) were satisfied with the intervention program and would recommend it to a friend or family member (67%). Most (79.2%) reported that the burden of testing with the study questionnaires was low. See Table 1 for Feasibility and Acceptability Criteria along with study results.

**Table 1:** Feasibility and acceptability criteria.

Proceed	Proceed with Protocol Amendments	Significant Amendments Required	CURRENT STUDY RESULTS
**Recruitment**			
n = 30 within 4 months	n ≥ 15 within 8 months	n < 10 within 8 months	n = 31 in 8 months
50% of eligible participants consent to participate	40% of eligible participants consent to participate	25% of eligible participants consent to participate	51.7% of eligible participants consent to participate
**Exercise program**			
60% of participants report exercise at least 3 times a week	40% of participants report exercise at least 3 times a week	25% of participants report exercise at least 3 times a week	Participants who reported exercising at least 3 times a week: Pre-op (n = 14): 71.4% Post-op (n = 12): 67.0%
**Content Acceptability**			
50% were satisfied with the program (Likert ≥ 4/5)	25% were satisfied with the program (Likert ≥ 4/5)	< 25% were satisfied with the program (Likert ≥ 4/5)	58% were satisfied with the program (Likert ≥ 4/5)
**Format Acceptability**			
50% reported being likely to recommend the program to a friend or family member	25% reported being likely to recommend the program to a friend or family member	< 25% reported being likely to recommend the program to a friend or family member	67% reported being likely to recommend the program to a friend or family member
**Follow Up**			
90% of participants followed up at the end of Prehab	50% of participants followed up at the end of Prehab	25% of participants followed up at the end of Prehab	83.9% of participants followed up at the end of Prehab
80% of participants followed up at 12-weeks post-op	50% of participants followed up at 12-weeks post-op	25% of participants followed up at 12-weeks post-op	77.4% of participants followed up at 12-weeks post-op
**Burden**			
75% of participants found the burden of completing questionnaires <3/10 (0 = no burden, 10 = most burden)	50% of participants found the burden of completing questionnaires <3/10 (0 = no burden, 10 = most burden)	25% of participants found the burden of completing questionnaires <3/10 (0 = no burden, 10 = most burden)	79.2% of participants found the burden of completing questionnaires <3/10 (0 = no burden, 10 = most burden)
**Adverse Events**			
No adverse event related to study activities	1 adverse event related to study activities	2 or more adverse events related to study activities	No adverse events related to study activities

### Exercise participation

Participants in the intervention group completed 24.6 (SD ± 8.3) exercise sessions during the 6-week pre-operative exercise phase and 23.9 (SD ± 6.9) sessions during the post-operative phase with minimal hernia symptoms. Most participants (71.4%) exercised at least three times per week pre-operatively and 67% of participants exercised at least three times per week post-operatively. There were no adverse events related to the intervention protocol. See Table 2.

**Table 2:** Exercise protocol implementation.

	Goal	Pre-op (n = 14)	Post-op (n = 12)
Sessions completed*	18	24.6 (±8.3)	23.9 (±6.9)
Participants who exercised at least 3x/week (%)	60.0%	71.4%	67.0%
Pain rating during exercise sessions* (/10)		0.7 (±1.3)	0.2 (±0.2)
Participants who did not track (no.)		2	2
Adverse events related to intervention	0	0	0
*Mean [± standard deviation (SD)].			

### Descriptive characteristics

The mean participant age was 49.4 years (SD ± 11.6), the mean duration of hernia was 72.8 weeks (SD ± 141.8), and 44.3% described themselves as regular exercisers. Participants were generally healthy with minimal co-existing medical conditions; any co-existing conditions were well-controlled. See Table 3 for baseline descriptive characteristics.

**Table 3:** Sample descriptive characteristics at baseline.

Characteristic	Total (n = 30)	Median, Min/Max	Control Group (n = 16)	Median, Min/Max	Intervention Group (n = 14)	Median, Min/Max
Age^*^ (years)	49.4 (±11.6)	51.0, 21.0-68.0	47.6 (±12.4)	47.5, 21.0-68.0	51.5 (±54.1)	52.5, 32.0-66.0
Sex, no. (%)						
Male	25 (83.3%)		13 (81.3%)		12 (85.7%)	
Female	5 (16.7%)		3 (18.8%)		2 (14.3%)	
BMI(kg/m²)^*^	26.1(±3.7)	26.0, 18.8-34.8	26.25 (±3.9)	26.0, 18.8-34.8	25.9 (±3.6)	25.25, 20.5-33.1
Ethnicity^ǂ^, no. (%)						
Canadian	19 (67.9%)		10 (62.5%)		9 (64.3%)	
Indigenous	2 (7.1%)		2 (12.5%)			
American	2 (7.1%)		1 (6.3%)		1 (7.1%)	
European - western	2 (7.1%)		1 (6.3%)		1 (7.1%)	
European - southern	1 (3.6%)				1 (7.1%)	
Asian - south	1 (3.6%)				1 (7.1%)	
South and Central American	1 (3.6%)				1 (7.1%)	
Duration of hernia^ǂ^^,^^*^ (weeks)	72.8 (±141.8)	30.0, 7.0-736.0	93.1 (±187.9)	28.0, 7.0-736.0	49.2 (±54.1)	35.0, 8.0-208.0
Regular exerciser, no. (%)	13 (43.3%)		7 (43.8%)		5 (35.7%)	
Active smoker, no. (%)	9 (30.0%)		7 (43.8%)		2 (14.3%)	
Job demands^ǂ^, no. (%)						
Light/Sedentary	10 (35.7%)		5 (31.3%)		5 (35.7%)	
Medium	10 (35.2%)		5 (31.3%)		5 (35.7%)	
Heavy	5 (17.9%)		4 (25.0%)		1 (7.1%)	
Very Heavy	3 (10.7%)		1 (6.3%)		2 (14.3%)	

* Mean [± standard deviation (SD)].

ǂ Missing: N = 2.

ª Missing: N = 1.

BMI (Body Mass Index)

### Results of performance-based testing

Of the 31 individuals enrolled in the study, 28 completed baseline assessment and functional testing. Three participants wore a hernia belt during testing. One participant had an active claim with the Workers’ Compensation Board of Alberta and had been assigned a lifting restriction of no greater than 15 kg prior to surgery, which was respected by the research team. During baseline performance-based testing, the mean weight lifted during the floor to waist lift test was 34.5 kg (SD ± 10.5), and 11 participants (39.3%) reached the study-implemented test ceiling (45 kg). Two participants reported mild hernia symptoms during the floor-to-waist lift testing. The mean pain rating during the floor to waist lift was 0.2 (SD ± 0.7) on the 10-point pain visual analogue scale. One participant was unable to complete the 5-minute rotation task due to hernia pain. The most problematic task was the abdominal endurance test, with 8 participants reporting hernia pain during this task. Twenty participants (71.4%) completed baseline functional testing with no hernia pain. See Table 4 for the results of the performance-based functional assessment.

**Table 4:** Results of performance-based testing.

	Baseline			Post-prehab		12 weeks post-op	
Task	Total (n = 28)	Control (n = 14)	Intervention (n = 14)	Control (n = 13)	Intervention (n = 13)	Control (n = 9)	Intervention (n = 12)
Floor-waist lift^*^ (kg)	34.5 (±10.5)	33.6 (±10.1)	35.7 (±11.2)	31.7 (±11.6)	31.7 (±14.7)	25.7 (±11.8)	35.5 (±10.9)
Pain during lift^*^ (/10)	0.2 (±0.7)	0.0 (±0.0)	0.4 (±0.9)	1.3 (±2.6)	1.4 (±2.6)	0.7 (±1.3)	0.0 (±0.0)
15 minutes standing^*^ (min)	15.0 (±0.0)	15.0 (±0.0)	15.0 (±0.0)	14.2 (±2.8)	15.0 (±0.0)	15.0 (±0.0)	15.0 (±0.0)
1 minute crouch^*^ (min)	60.0 (±0.0)	60.0 (±0.0)	60.0 (±0.0)	60.0 (±0.0)	60.0 (±0.0)	56.7 (±10.0)	60.0 (±0.0)
5-minute rotation^*^ (s)	292.2 (±41.2)	300.0 (±0.0)	284.4 (±58.3)	294.0 (±0.0)	290.0 (±24.5)	300.0 (±0.0)	300.0 (±0.0)
2-minute forward bend^*^ (min)	120.0 (±0.0)	120.0 (±0.0)	120.0 (±0.0)	112.7 (±26.3)	110.8 (±33.3)	107.8 (±24.4)	120.0 (±0.0)
Abdominal endurance test^*^ (s)	93.6 (±61.9)	90.6 (±67.7)	96.6 (±57.9)	82.9 (±60.7)	108.6 (±74.4)	74.8 (±57.9)	109.2 (±64.3)
Pain during abdominal endurance test^*^ (/10)	0.9 (±1.6)	0.9 (±1.7)	0.9 (±1.7)	0.8 (±2.1)	0.8 (±1.9)	0.3 (±1.0)	0.0 (±0.0)
30 second Sit to Stand^*^ (# of repetitions)	14.1 (±3.1)	13.5 (±3.1)	14.7 (±3.0)	14.2 (±2.8)	15.5 (±2.0)	12.2 (±3.3)	15.9 (±4.4)
Pain following testing^*^ (/10)	0.9 (±1.7)	0.9 (±1.8)	0.9 (±1.7)	1.9 (±2.9)	1.0 (±1.7)	0.6 (±1.1)	0.0 (±0.0)

* **Mean [± standard deviation (SD)].**

Six to eight weeks later during pre-op assessments, overall participant performance decreased slightly while pain levels increased slightly (See Table 4). The intervention group 1.0 (SD ± 1.7) reported less pain than the control group 1.9 (SD ± 2.9).

At 12-weeks post-op 3 participants (all belonging to the control group) completed final surveys but were unable to attend the final assessment due to work commitments, family emergency, or travel plans. There was a trend for participants in the intervention group to perform better overall than the control group during the final reassessment. They lifted more weight (35.5 kg (SD ± 10.9) versus 25.7 kg (SD ± 1.8)) and reported less pain after testing (see Table 4).

### Results of self-reported questionnaires

There was a trend for the intervention group to report less pain, lower scores on the PDI and CCS, and higher quality of life than the control group at 12-weeks post-operatively. See Table 5 for full self-reported data.

**Table 5:** Self-reported data.

	Baseline		Post-prehab		1-week Post-op		12-weeks Post-op	
	Control (n = 16)	Intervention (n = 14)	Control (n = 13)	Intervention (n = 13)	Control (n = 13)	Intervention (n = 12)	Control (n = 12)	Intervention (n = 12)
Pain level in past week^*^ (/10)	1.9 (±1.6)	3.0 (±2.0)	2.7 (±2.7)	1.7 (±1.3)	6.2 (±3.2)	5.5 (±2.7)	1.9 (±2.2)	0.8 (±1.1)
Pain level in past 24hrs^*^ (/10)	1.4 (±1.6)	2.2 (±1.5)	1.7 (±1.8)	1.4 (±1.4)	3.7 (±2.4)	3.8 (±2.6)	1.2 (±1.1)	0.5 (±0.7)
Worst pain level in past 24hrs^*^ (/10)	1.9 (±1.8)	2.9 (±2.2)	2.0 (±1.9)	1.8 (±1.8)	4.8 (±2.7)	4.3 (±2.4)	2.2 (±2.2)	0.5 (±0.7)
PDI rating^*^ (/70)	14.6 (±11.0)	16.7 (±13.0)	13.7 (±15.7)	9.7 (±7.0)	34.8 (±21.4)	25.4 (±15.7)	11.9 (±15.0)	1.8 (±2.7)
SF-12 Health Survey^*^ PCS-12 (Physical Score) MCS-12 (Mental Score)	44.3 (±10.2) 50.4 (±10.5)	41.7 (±8.0) 56.1 (±8.3)	44.7 (±10.0) 49.1 (±11.1)	43.9 (±8.8) 55.5 (±7.0)	36.7 (±7.2) 47.9 (±10.6)	33.9 (±9.2) 56.0 (±7.7)	44.1 (±8.1) 51.2 (±8.2)	53.5 (±2.7) 53.9 (±10.4)
Currently working, no. (%)	15 (93.8%)	13 (92.9%)	12 (92.3%)	12 (92.3%)	8 (61.5%)	4 (33.3%)	12 (100.0%)	9 (75.0%)
Currently performing regular work tasks, no. (%)	12 (80.0%)	12 (92.3%)	10 (83.3%)	11 (91.7%)	5 (62.5%)	4 (100.0%)	9 (75.0%)	9 (100.0%)
CCS^*^ (post-op only)	n/a	n/a	n/a	n/a	48.0 (±21.5)	38.5 (±20.5)	20.0 (±21.1)	2.8 (±3.6)

* Mean [± standard deviation (SD)].

PDI (Pain Disability Index).

SF-12 (Short Form-12 Health Survey).

PCS-12 (Physical Component Summary).

MCS-12 (Mental Component Summary).

CCS (Carolinas Comfort Scale).

### Adverse events

As outlined in Table 1, there were no adverse events relating to study activities. There were two participants with hernia symptom flare-ups severe enough to warrant a referral to the surgeon. One participant had severe pain (< 6/10) at the post-prehab follow-up. This participant was randomized to the intervention group but did not regularly complete the assigned exercise program. His hernia flare-up was related to his physical occupation and was cleared safe by the surgeon and did not require emergent surgical intervention. The other participant was in the control group and re-injured himself at work after returning to work following his IHR. He was referred to the surgeon who confirmed a hernia recurrence.

## Discussion

### Feasibility

The recruitment rate was 51.7%, which was slightly above our pre-determined acceptable rate of 50%. Two items that warrant protocol amendments included the proportion of followed-up participants at the end of prehab, and at the end of the 12-week follow-up period. Both were slightly under our pre-determined rates for acceptability. All other feasibility criteria were fully met. Feasibility was complicated by the COVID-19 pandemic, with health and safety guidelines fluctuating and several cancellations occurring due to illness. With the pandemic also affecting many people’s workplaces, we experienced some difficulty with recruitment due to individuals’ inability to take time off to attend assessments in-person. Only including people who worked full-time as part of the inclusion criteria became challenging as individuals were unable to commit to assessments due to work commitments, or else they were not eligible since they worked seasonally, part time, or had recently retired. This combination of work-related barriers and being unable to attend in-person assessments contributed to participant dropout and loss to follow-up. We ultimately expanded our inclusion criteria to include other than full-time work, which is recommended when designing future trials.

### Clinical assessments

While adequate numbers were enrolled and assessed in this pilot study, our assessment protocol likely did not adequately capture functional capacity as many participants performed to the ceiling or maximal levels for the various performance tests. This likely means that amendments are needed in future studies. Minimal hernia pain was reported during in-person performance-based testing and none of the 11 participants that reached the floor-to-waist lift ceiling during baseline testing reported hernia symptoms during the task. Interestingly, the floor-to-waist lift – a common activity that many patients with inguinal hernia are advised by their primary care providers to avoid – produced minimal symptoms and was performed to high levels. This brings into question the usefulness of lifting restrictions that are commonly recommended for people with inguinal hernia.

These trends were similar for post-prehab and 12-week post-op reassessments. Participants reported slightly increased pain at the post-prehab assessment, which is expected as typically inguinal hernia becomes more uncomfortable over time [29]. At the final 12-week post-op in-person testing, half (6/12) of participants in the intervention group reached the floor-to-waist lift ceiling, while only one (out of 9) participant in the control group reached the ceiling. While no statistical comparisons were made, it is encouraging to note that the intervention group generally reported less pain, disability, hernia symptoms, and improved quality of life at 12-weeks post-op. This provides support for further evaluation of our intervention through a larger RCT.

## Limitations

The possibility of selection bias was high in this pilot study due to working with one surgeon who was involved in planning the study and responsible for screening eligible patients for the study. It is possible that rather than telling every screened patient about the study, only those who were seen as a good fit were referred. This may have led to the high levels of reported fitness and exercise participation among our sample. Some of this bias could be eliminated in a larger study by opening recruitment to a wider population, recruiting from additional surgeons’ clinics, and using posters and self referral of interested patients. However, working with only one surgeon ensured that all patients underwent a similar screening process and surgical procedure with surgical skill level remaining constant. This decreased the variability that would have come with multiple surgeons of various skill levels using differing procedures to complete the IHR. Using one surgeon, one assessing physical therapist, and one exercise professional to administer the intervention kept inter-examiner/practitioner variability low.

It was not possible to blind the research participants or the assessor to the exercise intervention. Efforts were made to keep observer bias low by blinding the assessing physical therapist to which group participants were randomized. Yet despite attempts to minimize interaction with the assessor to reduce the risk of revealing what group participants were in, it is likely that extended discussion with the participants and marked improvements in lifting ability and technique revealed group allocation.

Coming for in-person assessments was the main barrier to participant retention. While 24 participants completed all final self-reported assessments, only 21 participants completed the final 12-week post-op assessment. This final number of participants is lower than the sample size recommended by Lewis et al. [16] as being sufficient for indicating acceptable fidelity. Participant dropout rates should be carefully considered when recruiting for a larger study. Participants in the control group were more likely to drop out than the intervention group, which should be investigated further as a previous study found that randomization to a prehabilitation group led to higher rates of drop out [10]. Anecdotally, several of our participants voiced disappointment that they were not randomized to the intervention group, which may explain the higher dropout rates in the control group.

### Considerations for further study

While feasibility criteria were met to justify a larger more definitive study, addressing some of the encountered challenges will further increase feasibility. Recruitment rates could likely be improved by expanding to recruit more participants from multiple surgeons (with similar levels of expertise and who use similar techniques) and expanding inclusion criteria regarding work status. Future studies should consider using self-report measures only to ease participant testing burden. The use of a performance-based functional assessment protocol should also be reconsidered since participants performed very well on the test items, with several reaching the ceiling for most items. In-person assessment also posed a barrier to follow-up. We suggest future studies rely on self-reported questionnaires, which are easier to administer and present less burden to participants. This may also increase recruitment as it would not require patients to schedule time away from work, which was the most common reason that individuals opted out of the study. A study with remote delivery of the intervention and no in-person requirements would be easier to distribute, reach a higher number and variety of patients, and eliminate some sources of potential bias.

## Conclusion

The goal of our pilot study was to determine the feasibility of a perioperative rehabilitation program for patients undergoing IHR, and to provide meaningful information to inform a larger study. Participant recruitment, adherence, and retention appeared adequate and indicate a larger study would be feasible. We recommend the measurement protocol be changed to rely on self-report questionnaires rather than performance-based tests. With remote delivery of the intervention and assessment, a larger study could access more patients and be easier to conduct, while generating meaningful, relevant information to help guide clinical recommendations and improve outcomes for individuals undergoing IHR.

## Supporting information

S1 Appendix A Perioperative rehabilitation activity and data collection protocol for inguinal hernia repair.
(PDF)

S2 Appendix B Perioperative rehabilitation protocol for inguinal hernia repair.
(PDF)

S3 Appendix C Hernia educational videos.
(PDF)

S1 Text CONSORT Checklist. (DOCX)

S1 File (PDF)
