# Supplementary material for: A pilot randomized controlled trial examining the feasibility of perioperative rehabilitation for inguinal hernia repair surgery
Source: PLoS One. 2025 May 22;20(5):e0324907. doi: 10.1371/journal.pone.0324907 (PMC12097709; doi:10.1371/journal.pone.0324907)
Supplement: S1 Appendix A — Perioperative rehabilitation activity and data collection protocol for inguinal hernia repair. (PDF) [file pone.0324907.s001.pdf]

Appendix A:

Perioperative Rehabilitation Activity and Data Collection Protocol for Inguinal Hernia Repair

(Intervention group)

Before the operation – Approximately 12-hour commitment

Timing	*Activity*	*Measures Collected*
8 weeks pre-op	Recruitment and consent process.<br>In-person meeting: baseline data collection (partially done online using REDCAP if possible) and physical assessment (1 hour)<br><br>Deliver Prehab for Hernia video to intervention group	Demographic and Work Status Questionnaire<br>Numerical Pain Scales<br>Pain Disability Index<br>SF-12 Health Survey<br>Work-Related Functional Testing
6 weeks pre-op	In-person meeting: start 6-week pre-op exercise protocol (1 hour)	Adherence<br>Adverse Events
Weekly between weeks 6 until operation	Patient to do exercises and education sessions on their own at home 5x/week in 15–20-minute sessions<br>(1.5 hours per week x 6 weeks = 9 hours)<br><br>Virtual follow-ups 1x/week to progress as needed, monitor adherence and provide guidance and motivation (included in exercise time above)	Adherence<br>Adverse Events
1 week pre-op	In-person meeting: Last visit before surgery, interim data collection and physical assessment (1 hour)<br><br>Deliver pain management video and surgery information video to both control and intervention groups<br>Deliver post surgery activity video to intervention group	Work Status Questionnaire<br>Numerical Pain Scales<br>Pain Disability Index<br>SF-12 Health Survey<br>Work-Related Functional Testing

After the operation - Approximately 18-hour commitment

Timing	*Activity*	*Measures Collected*
1 week post-op	Electronic survey using REDCAP for pain/complications (Done online, 15 minutes)	Numerical Pain Scales<br>Pain Disability Index<br>Carolinas Comfort Scale questionnaire

2 weeks post-op	In-person follow-up with surgeon	
3 weeks post-op	In-person meeting: Start 6-week post-op exercise protocol (1 hour)	Adherence<br>Adverse Events
Weekly between weeks 3 to 12	Patient to do exercises on their own at home 5x/week in 15–20-minute sessions<br>(1.5 hours per week x 10 weeks = 15 hours)<br><br>Virtual follow-ups 1x/week to progress as needed, monitor adherence and provide guidance and motivation<br><br>Weekly monitoring for adverse effects included in virtual follow-ups	Adherence<br>Adverse Events
4 weeks post-op	In-person surgeon follow-up (as part of usual clinical practice)	Ultrasound if needed
9 weeks post-op	Virtual check-in, return to work/activity advice (30 minutes)	Adherence<br>Adverse Events
12 weeks post-op	Final in-person assessment<br>(1 hour)	Numerical Pain Scales<br>Pain Disability Index<br>Work Status Questionnaire<br>SF-12 Health Survey<br>Carolinas Comfort Scale questionnaire<br>Work-Related Functional Testing<br><br>Feedback and Satisfaction Survey
