# Supplementary material for: A pilot randomized controlled trial examining the feasibility of perioperative rehabilitation for inguinal hernia repair surgery
Source: PLoS One. 2025 May 22;20(5):e0324907. doi: 10.1371/journal.pone.0324907 (PMC12097709; doi:10.1371/journal.pone.0324907)
Supplement: S2 Appendix B — Perioperative rehabilitation protocol for inguinal hernia repair. (PDF) [file pone.0324907.s002.pdf]

Appendix B:

Perioperative Rehabilitation Protocol for Inguinal Hernia Repair

Exercise protocol (Intervention group)

General goals for having the intervention group complete exercise prior to and following inguinal hernia repair surgery is to strengthen the abdominal wall and improve general physical resilience in this population. It is assumed that many of the individuals in this population have a low physical fitness level to begin with and that any exercise would bring them benefit, however for the purpose of this study exercises have been chosen that have components of abdominal strengthening, functional strength, and ergonomic conditioning.

Exercises will be initially introduced and taught by a physical therapist or other qualified exercise professional 6 weeks before the operation, and gradually reintroduced after the operation starting at three weeks post-op after being cleared by the surgeon. Initial introduction of exercise following surgery will begin with gentler activities such as diaphragmatic breathing, transverse abdominis activation, and simple bridging, and over six weeks of supervision progress weekly to more complicated and functional exercises.

Instruction will include attention to breath coordination during strengthening activities in order to avoid valsalva maneuver and increasing intrathoracic pressure. Participants will be encouraged to spend approximately 15-20 minutes daily completing their exercise routine with the goal to see exercises completed at least 5x/week. Exercise intensity will be adapted according to individual ability and presence of pain and will be delivered and tracked online using physitrack.com.

See following tables for example exercise outline.

6 weeks before the operation – full program instruction

Exercise	*Basic/Starting*	*Progression*
Diaphragmatic breathing	Instruction for breathing into three sections of the lungs: collar bones, ribs, belly.<br>3 cycles of 3 breaths.	
Transverse abdominis (TA) activations	Instruct activation of the transverse abdominis muscle using breath cueing.	Add pelvic floor activation.<br>Add knee lifts or heel slides.<br>Add 90° leg hold.
Bridging	Raise hips off the mat, squeeze glutes.	Incline bride.<br>One-leg bridge
Bird-dogs (4-point progression)	May begin by using only one limb at a time.	Opposite arm and leg extend.
Dead-bugs	May begin by using only one limb at a time, having knees bent.	Opposite arm and leg extend; straight legs.
Chair squat	Weight-bearing.<br>May begin with sit to stand if necessary.	Add theraband to increase glute med. firing.<br>Add weight
Push up progression	Begin with wall push ups	Progress to counter/knee/floor push ups
Floor to waist lift	Weight-bearing.<br>Begin with no weight - 10 lbs	Increase weight according to job demands.

After the operation – gradual program implementation

Timing	*Exercise*	*Progression (as tolerated)*
3 weeks post-op	Diaphragmatic breathing<br>TA activations<br>Bridging	
4 weeks post op	Diaphragmatic breathing<br>TA activations<br>Bridging<br>+Bird-dogs (one limb)<br>+Dead-bugs (one limb)<br>+Sit to stands	Add:<br>-knee lift to TA activations<br>-increase lift and hold on bridging
5 weeks post-op	Diaphragmatic breathing<br>TA activations	Add:<br>-body weight chair squats

	Bridging<br>Bird-dogs (one limb)<br>Dead-bugs (one limb)<br>(Chair) squats<br>+Wall push ups<br>+Floor to waist lifts (10lbs)	-catch up on progressions as appropriate
6 weeks post-op	Diaphragmatic breathing<br>TA activations<br>Bridging<br>Bird-dogs<br>Dead-bugs<br>Chair squats (with band)<br>Push up progression<br>Floor to waist lifts (15 lbs)	Add:<br>-opposite arm/leg to bird dogs and dead bugs<br>-band to squat<br>-add weight to floor to waist lift
7 weeks post-op	Diaphragmatic breathing<br>TA activations<br>(One leg) Bridging<br>Bird-dogs<br>Dead-bugs<br>Chair squats<br>Push up progression<br>Floor to waist lifts (20 lbs)	Add:<br>-one-leg bridge<br>-increase weight on floor to waist lift
8 weeks post-op	Diaphragmatic breathing<br>TA activations<br>(one leg) Bridging<br>Bird-dogs<br>Dead-bugs<br>Squats<br>Push ups<br>Floor to waist lifts (20 lbs)	Add:<br>-increase weight on floor to waist lift<br>-progress push ups as tolerable
9 weeks post-op	Diaphragmatic breathing<br>TA activations<br>(one leg) Bridging<br>Bird-dogs<br>Dead-bugs<br>Squats<br>Push ups<br>Floor to waist lifts (30 lbs)	Add:<br>-increase weight on floor to waist lift
