# Supplementary material for: A pilot randomized controlled trial examining the feasibility of perioperative rehabilitation for inguinal hernia repair surgery
Source: PLoS One. 2025 May 22;20(5):e0324907. doi: 10.1371/journal.pone.0324907 (PMC12097709; doi:10.1371/journal.pone.0324907)
Supplement: S3 Appendix C — Hernia educational videos. (PDF) [file pone.0324907.s003.pdf]

Appendix C:

<u>Hernia Educational Videos</u>

Pain Management Video: https://www.youtube.com/watch?v=M1Yri-RCtYw

Hernia Surgery Video: https://youtu.be/JQA2RjyJ19c

Prehabilitation Video: https://www.youtube.com/watch?v=gpiLpNJIPh0

Post-surgery Rehabilitation Video: https://www.youtube.com/watch?v=bCoHXe7R-4U
