# Supplementary material for: A pilot randomized controlled trial examining the feasibility of perioperative rehabilitation for inguinal hernia repair surgery
Source: PLoS One. 2025 May 22;20(5):e0324907. doi: 10.1371/journal.pone.0324907 (PMC12097709; doi:10.1371/journal.pone.0324907)
Supplement: S1 File — (PDF) [file pone.0324907.s005.pdf]

Date: March 12, 2021 2:59:16 PM Print Close

1.1 Study Identification

*All questions marked by a red asterisk * are required fields. However, because the mandatory fields have been kept to a minimum, answering only the required fields may not be sufficient for the REB to review your application.*

Please answer all relevant questions that will reasonably help to describe your study or proposed research.

1.0 * **Short Study Title** *(restricted to 250 characters)*:
Peri-Operative Rehab Program for Inguinal Hernia Repair Surgery

2.0 * **Complete Study Title** *(can be exactly the same as short title)*:

A Study Evaluating the Feasibility of a Peri-Operative Rehabilitation Program for Inguinal Hernia Repair Surgery to Reduce Risk of Post-Surgical Pain

3.0 * **Select the appropriate Research Ethics Board (***Detailed descriptions are available at here***):**
Health Research Ethics Board - Health Panel

4.0 * **Is the proposed research:**
Unfunded

5.0 * **Name of local Principal Investigator:**
Douglas Gross

6.0 * **Type of research/study:**
Faculty/Academic Staff

7.0 **Investigator's Supervisor***(required for applications from undergraduate students, graduate students, post-doctoral fellows and medical residents to REBs 1 & 2. HREB does not accept applications from student PIs)*:

8.0 **Study Coordinators or Research Assistants:** People listed here can edit this application and will receive all email notifications for the study:

Name	Employer
Robert Piper	Student

9.0 **Co-Investigators:** People listed here can edit this application and will receive email notifications *(Co-investigators who do not wish to receive email, should be added to*

the study team below instead of here).
If your searched name does not come up when you type it in the box, the user does not have the Principal Investigator role in the online system. Click the following link for instructions on how to Request an Additional Role.

Name	Employer
Omar Farooq	MH Surgery

10.0 **Primary Admin Contact** *(a member of study team)*:

11.0 **Study Team:** *(co-investigators, supervising team, and other study team members) - People listed here cannot view or edit this application and do not receive email notifications.*

Last Name	First Name	Organization	Role/Area of Responsibility	Phone	Email
Macedo	Luciana	McMaster University	Co-Principal Investigator		macedol@mcmaster.ca
Bostick	Geoff	Department of Physical Therapy	Co-investigator		bostick@ualberta.ca
Durand-Moreau	Quentin	Division of Preventive Medicine	Co-Investigator		durandmo@ualberta.ca
Piper	Robert Chad	Department of Physical Therapy	Graduate Student Co-investigator		robertch@ualberta.ca
Shologan	Anna	AHS	Co-investigator		akshologan@gmail.com

1.2 Additional Approval

1.0 * **Departmental Review:** ***Please note only ONE Department Review is required. Please ensure that this section reflects only the PRIMARY Department of the study PI.***

RM Physical Therapy

2.0 **Internal Review** *(If the Principal Investigator is in the Department of Medicine complete the Department of Medicine Request for Internal Approval form and upload it to the "Documentation" section of this application under item 11.0 "Other Documents". Note that all fields in the form are required. The form is available at here)*:

1.4 Conflict of Interest

1.0 * **Are any of the investigators or their immediate family receiving any personal remuneration (including investigator payments and recruitment incentives but excluding trainee remuneration or graduate student stipends) from the funding of this study that is not accounted for in the study budget?**

○ Yes ● **No**

2.0 * **Do any of investigators or their immediate family have any proprietary interests in the product under study or the outcome of the research including patents, trademarks, copyrights, and licensing agreements?**

○ Yes ● **No**

3.0 * **Is there any compensation for this study that is affected by the study outcome?**

○ Yes ● **No**

4.0 * **Do any of the investigators or their immediate family have equity interest in the sponsoring company? (This does not include Mutual Funds)**

○ Yes ● **No**

5.0 * **Do any of the investigators or their immediate family receive payments of other sorts, from this sponsor (i.e. grants, compensation in the form of equipment or supplies, retainers for ongoing consultation and honoraria)?**

○ Yes ● **No**

6.0 * **Are any of the investigators or their immediate family, members of the sponsor's Board of Directors, Scientific Advisory Panel or comparable body?**

○ Yes ● **No**

7.0 * **Do you have any other relationship, financial or non-financial, that, if not disclosed, could be construed as a conflict of interest?**

○ Yes ● **No**

Please explain if the answer to any of the above questions is Yes:

Important

If you answered YES to any of the questions above, you may be asked for more information.

1.5 Research Locations and Other Approvals

1.0 * **List the locations of the proposed research, including recruitment activities. Provide name of institution, facility or organization, town, or province as applicable**

Fort Saskatchewan Community Hospital - Dr. Farooq's clinic
Corbett Hall, University of Alberta

2.0 * **Indicate if the study will use or access facilities, programmes, resources, staff, students, specimens, patients or their records, at any of the sites affiliated with the following** *(select all that apply):*

Alberta Health Services Institutions and Facilities

List all health care research sites/locations:

Fort Saskatchewan Community Hospital - Dr. Farooq's clinic

3.0

Multi-Institution Review

* **3.1 Has this study already received approval from another REB?**

○ Yes ● **No**

4.0 **If this application is closely linked to research previously approved by one of the University of Alberta REBs or has already received ethics approval from an external ethics review board***(s)*, **provide the study number, REB name or other identifying information. Attach any external REB application and approval letter in the Documentation Section – Other Documents.**

2.1 Study Objectives and Design

1.0 * **Provide a lay summary of your proposed research which would be**

understandable to general public

Inguinal hernia repair is one of the most common surgeries, with more than 20 million performed globally each year. It is estimated that approximately 15% of patients undergoing inguinal hernia repair will experience persistent post-surgical pain that could last months to years. Evidence from related procedures indicates that better surgical preparation through pre-operative exercise and education (i.e. Prehabilitation) followed by ongoing post-surgical rehabilitation leads to more rapid recovery, return to activities and lower likelihood of persistent post-surgical pain. We will determine the feasibility of a peri-operative rehabilitation program (pre- and post-surgery) and our study protocol for patients undergoing inguinal hernia repair surgery. We hypothesize that: 1) our peri-operative intervention will be feasible and safe to undertake within a clinical setting; 2) we will enrol adequate numbers to justify a larger trial; and that 3) our outcome measurement protocol will provide meaningful information with high response rate and low attrition after 3 months.

Implications

This study is novel and timely given the current healthcare focus on improving strategies for the prevention of chronic pain. The obvious potential benefits for patients with inguinal hernia is the development of a program with the potential to improve post-surgical outcomes and reduce likelihood of post-surgical pain. Our findings will inform a larger, multicenter clinical trial to evaluate this intervention approach in patients undergoing inguinal hernia repair.

2.0 * **Provide a full description of your research proposal outlining the following:**

- **Purpose**
- **Hypothesis**
- **Justification**
- **Objectives**
- **Research Method/Procedures**
- **Plan for Data Analysis**

Purpose

The aim of the proposed project is to determine the feasibility of a peri-operative rehabilitation program (pre- and post-surgery) and our study protocol for patients undergoing inguinal hernia repair surgery. We will answer the following question: **Is a study of a peri-operative rehabilitation program feasible in terms of recruitment rate, treatment adherence, timelines, data collection procedures, patient follow-up and resources required?**

Hypothesis

We hypothesize that: 1) our peri-operative intervention will be feasible and safe to undertake within a clinical setting; 2) we will enrol adequate numbers to justify a larger trial; and that 3) our study protocol will provide meaningful information with high response rate and low attrition after 3 months.

Justification

Inguinal hernia repair is one of the most common surgeries performed globally, with more than 20 million performed each year.[1] Not only does this huge number of surgeries have a substantial direct economic burden on healthcare systems, there is also indirect impact from time off work and decreased productivity due ongoing post-surgical pain requiring extended time spent on modified duties. It is estimated that approximately 15% of patients undergoing inguinal hernia repair will experience persistent post-surgical pain that could last months to years.[2, 3] Furthermore, current clinical guidelines for return to work and activity after inguinal hernia repair are inconsistently informed by evidence, highly variable, and outdated. [4] Forbes et al (2012) found that the average patient undergoing inguinal hernia repair experiences more than 40 days of short-term disability despite indications that earlier return to activity is safe (i.e. will not lead to repair failure) and likely beneficial for reducing chronic pain and disability.[4]

Evidence from related procedures indicates that better surgical preparation through pre-operative exercise and education (i.e. Prehabilitation) followed by ongoing post-surgical rehabilitation leads to more rapid recovery, return to activities and lower likelihood of persistent post-surgical pain. [5] Prehabilitation was first described in the 1940’s when the British Army developed a prehabilitation program as part of an experiment to increase the quality of recruits.[6]The concept of prehabilitation gained traction within the medical community when Topp et. al.[7] and Ditmyer et. al.[8]promoted a theoretical model of prehabilitation, positing that patients who participate in presurgical exercise with the goal of improving functional capacity may experience more rapid postoperative recovery than patients who remain physically inactive through the preoperative period.[6] Prehab has also been shown to increase self-efficacy,[9] a moderator of pain catastrophizing and fear avoidance beliefs, that are important factors linked to the pain experience of those with persistent pain. We propose that this theoretical model can be extrapolated to inguinal hernia repair and theorize that prehabilitation will increase patients’ preoperative self-efficacy, allowing them to more rapidly regain abilities, subsequently shortening duration of modified duties and decreasing likelihood of persistent post-surgical pain.

Little information is available regarding the use of prehabilitation in the context of inguinal hernia repair. There have been numerous studies looking at prehabilitation in orthopedics, cardiovascular surgery, and prior to major abdominal surgeries, but limited studies for inguinal hernia repair.[5] A randomized control study by Liang et. al. examined the impact of prehabilitation on ventral hernia recurrence and post-operative complications.[10] They concluded that patients undergoing prehabilitation have a higher likelihood of being hernia-free and complication-free 30 days postoperatively. Notably, this study is limited in that only obese patients were included, and outcomes only included recurrence and complications. We were unable to find studies examining the impact of prehabilitation on recovery time, post-surgical pain, and return to activity after inguinal hernia repair. However psychosocial factors such as pain catastrophizing appear to be important predictors.[11] Typical practice guidelines often recommend limiting activity for at least 3 months to avoid re-rupture.[4] However, these guidelines are based on expert opinion due to a lack of quality research and can pose a risk in building unhelpful beliefs about pain, fear of movement and poor coping strategies.[12] Research is needed to inform practice guidelines and return-to-activity recommendations.

Objectives

We will determine if our program and the study protocol are feasible for use on a group of patients with inguinal hernia undergoing surgical repair.

Research Methods/Procedures

Design - We will conduct a pilot randomized controlled trial. Specifically, this project will inform the development and testing of a specific exercise and education program, tailored to each patient's individual needs and conducted before and after hernia repair surgery, aimed at reducing likelihood of chronic post-surgical pain while doing minimal harm.

Sample - We will recruit patients referred to general surgery for an elective inguinal hernia repair within Alberta Health Services – Fort Saskatchewan Community Hospital (Dr. Farooq's clinic). Patients meeting the inclusion criteria will be randomized 1:1 to either undergo 6 weeks of prehabilitation exercises + post-surgical follow-up or else usual care. Inclusion criteria include: 1) scheduled to undergo first-time inguinal hernia repair surgery after a physical examination identified signs and symptoms consistent with inguinal hernia (direct or indirect hernia); 2) willingness to participate in a targeted perioperative exercise program; 3) 18+ years of age; 4) no medical contraindications to participation in exercise; and 5) employed full-time and required to lift at least 10kg at work. Specific c*ontra-indications that will be screened for include associated medical conditions that preclude exercise. This includes uncontrolled medical conditions including diabetes, hypertension, vertigo, congestive heart failure, chronic obstructive pulmonary disease, intra-abdominal ascites, or pre-existing malnutrition. We will also screen for previous recurrence of hernia, use of narcotics, bone and joint conditions of the spine or extremities, or history of other abdominal surgeries, which will be exclusionary criteria for the study.* Exclusion criteria include: 1) previous inguinal hernia; and 2) Body Mass Index >35 since morbidly obese patients experience more surgical complications.[13]

Sample Size - Since this is a pilot study evaluating feasibility of our intervention and evaluation protocols, we will aim to enrol 30 patients. We will randomize 15 to both the intervention and control groups. Thirty participants has been deemed acceptable to yield confidence intervals whose lower limits can help define the range of plausible values for a subsequent power analysis.[14] This will also provide adequate estimates of enrolment, follow-up rates, information on timing, and variance in our measurement protocol to inform a subsequent randomized controlled trial of the intervention.

*Data Collection Procedures – Activities and timing of data collection are outlined in the attached document entitled '***Perioperative Rehabilitation Activity and Data Collection Protocol for Inguinal Hernia** ' We will enrol patients undergoing inguinal hernia repair surgery. Participation will be voluntary, and patients will be informed of the study 8 weeks before their scheduled surgery. Once scheduled and enrolled, patients will be randomized by a research team member using Redcap. Schedule will be developed by a statistician not involved in the randomization. Participants in the intervention group will be referred to the University of Alberta's Student Physical Therapy clinic where they will undertake the pre-operative exercise and education program. The education program will include self-management, expectations about the surgical and recovery process as well as guidelines for post-operative exercises. After surgery, participants in the intervention group will undergo a clinical evaluation after surgery, with the physiotherapists who will guide post-operative exercises and modify as

needed. The exercises will avoid all contraindications in the acute post-surgical period, including no lifting >10kg for the first 4 weeks. The study will not impact scheduling of surgery or typical procedures. Participants in the intervention group will be booked for a short assessment with the general surgeon, remotely, to evaluate progress.

COVID-19 Implications - Since inguinal hernia repair operations have continued to be conducted despite the pandemic and physiotherapy clinics are operating following public health recommendations, unless these interventions are out on hold we will conduct this study within the typical clinical care pathway. After an initial in-person evaluation where masks will be worn and all exercise and testing equipment is sterilized after use, all interventions will be conducted in accordance with the COVID-19 specific requirements from Public Health authorities. Telehealth options for the interventions will be available.

Intervention Description - The perioperative rehabilitation program will include an established set of structured exercises aimed at core strengthening and individualized activity-based functional training in pre- and post- operative stages. We will follow a traditional occupational rehabilitation approach, focusing exercise training on activities that the patient is experiencing difficulty with due to the hernia. The exercise program is described in more detail in the attached '**hernia exercise protocol**' document. The program will be delivered by a physiotherapist and a trained exercise therapist. *This includes the sessions that will be done in person as well as those that will be done virtually. Education that will be provided includes basic information about inguinal hernia and the surgical repair technique, what to expect in the days and weeks following surgery, advice for post-operative recovery, importance of activity and exercise to recovery, pain coping strategies and techniques for reducing risk of hernia recurrence. Education will be provided in person by the surgeon and study team, as well as through YouTube videos that provide more standardized information. Education that will be provided includes basic information about inguinal hernia and the surgical repair technique, what to expect in the days and weeks following surgery, advice for post-operative recovery, importance of activity and exercise to recovery, pain coping strategies and techniques for reducing risk of hernia recurrence.*

Measures – In additional to demographic and occupational information, we will collect feasibility and clinical outcomes.Feasibility outcomes are: 1) recruitment rate expressed as the average number of patients referred to the study per week and the number of participants enrolled in the program; 2) patient adherence to the program; 3) acceptability of program content; 4); acceptability and compliance with study questionnaires; 5) follow-up rates; and 6) serious adverse events. A priori criteria for the feasibility outcomes are presented in the Appendix.

Clinical outcomes will be collected at baseline, before surgery, and following the post-surgical intervention by a researcher not involved in the delivery of the intervention. Persistent post-surgical will be defined as pain lasting longer than 3 months post-surgery as per the IASP definition.[15] We will measure pain intensity ratings on a 0-10 Numeric Pain Scale. We will also measure pain-related disability using the Pain Disability Index and SF-12 measures. Lastly, after the operation we will administer the hernia-specific Carolinas Comfort Scale (CCS).[16] The CCS is a widely used hernia-specific patient reported outcome measure that focuses on mesh-related symptoms.[17] We will also measure quality of life and factors linked

to chronic pain such as coping strategies, catastrophizing and Kinesiophobia.

Safety and adverse effects – At the weekly sessions, we will as about both exercise completion as well as any symptoms or adverse events experienced. These will be documented. Any post-surgical complications severe enough to warrant referral to the surgeon or ultrasound imaging will also be tracked. Since the ultrasounds are only being done to rule out hernia recurrence as part of routine clinical care, this will be done and interpreted by a radiologist. All patients will also undergo a consult with the surgeon at 1-week, 1-month, and 3-month follow-up as per usual practice.

Plan for Data Analysis

A sample size of 30 participants will yield confidence intervals whose lower limits can help define the range of plausible values for a subsequent power analysis program. This will guarantee the timely and on-budget completion of the study. All analyses will be conducted using STATA 14.2. Descriptive analysis will be used to report feasibility outcomes (e.g. recruitment rates, adherence, etc.) and identify trends of clinical outcomes. Feasibility targets will be evaluated as per the Appendix.

See Attached Study Protocol for the full list of references.

3.0 **Describe procedures, treatment, or activities that are above or in addition to standard practices in this study area** *(eg. extra medical or health-related procedures, curriculum enhancements, extra follow-up, etc)***:**
The Prehab program discussed above that we are studying is in addition to the standard surgical practices in this study area.

4.0 **If the proposed research is above minimal risk and is not funded via a competitive peer review grant or industry-sponsored clinical trial, the REB will require evidence of scientific review. Provide information about the review process and its results if appropriate.**
This project is under review for a Michael G. DeGroote Institute for Pain Research and Care Seed Grants

5.0 **For clinical trials, describe any sub-studies associated with this Protocol.**
Not applicable.

2.2 Research Methods and Procedures

Some research methods prompt specific ethical issues. The methods listed below have additional questions associated with them in this application. If your research does not involve any of the methods listed below, ensure that your proposed research is adequately described in Section 2.1: Study Objectives and Design or attach documents in the Documentation Section if necessary.

1.0 * **This study will involve the following***(select all that apply)*
Surveys and Questionnaires (including internet surveys)

NOTE 1: Select this ONLY if your application SOLELY involves a review of paper charts/electronic health records/administrative health data to answer the research question. If you are enrolling people into a study and need to collect data from their health records in addition to other interventions, then you SHOULD NOT select this box.

NOTE 2: Select this option if this research ONLY involves analysis of blood/tissue/specimens originally collected for another purpose but now being used to answer your research question. If you are enrolling people into the study to prospectively collect specimens to analyze you SHOULD NOT select this box.

2.9 Surveys and Questionnaires (including Online)

1.0 **How will the survey/questionnaire data be collected (i.e. collected in person, or if collected online, what survey program/software will be used etc.)?**
If participants are unable to complete study questionnaires on REDCAP, we will provide paper copies. But this will not be based on preference. We will complete as much as possible using REDCAP to minimize contact due to COVID-19.

2.0 **Where will the data be stored once it's collected (i.e. will it be stored on the survey software provider servers, will it be downloaded to the PI's computer, other)?**
Data will be downloaded and stored on the Co-Principal Investigators' computers.

3.0 **Who will have access to the data?**
The Co-Principal investigators and any graduate students supervised by the Co-PIS working on this project will have access to the data.

4.0 **If you are using a third party research tool, website survey software, transaction log tools, screen capturing software, or masked survey sites, how will you ensure the security of data gathered at that site?**

3.1 Risk Assessment

1.0 * **Provide your assessment of the risks that may be associated with this research:**
Minimal Risk - research in which the probability and magnitude of possible harms implied by participation is no greater than those encountered by participants in those aspects of their everyday life that relate to the research (TCPS2)

2.0 * **Select all that might apply:**

Description of Possible Physical Risks and Discomforts

Yes	Participants might feel physical fatigue, e.g. sleep deprivation
Yes	Participants might feel physical stress, e.g. cardiovascular stress tests
Yes	Participants might sustain injury, infection, and intervention side-effects or complications
Yes	The physical risks will be greater than those encountered by the participants in everyday life

Possible Psychological, Emotional, Social and Other Risks and Discomforts

Possibly	Participants might feel psychologically or emotionally stressed, demeaned, embarrassed, worried, anxious, scared or distressed, e.g. description of painful or traumatic events
Possibly	Participants might feel psychological or mental fatigue, e.g intense concentration required
Possibly	Participants might experience cultural or social risk, e.g. loss of privacy or status or damage to reputation
No	Participants might be exposed to economic or legal risk, for instance non-anonymized workplace surveys
Possibly	The risks will be greater than those encountered by the participants in everyday life

3.0 * **Provide details of all the risks and discomforts associated with the research for which you indicated YES or POSSIBLY above.**

Since the Prehab program involves physical exercise by the participants, there is an inherent element of physical risk. This may involve fatigue, tiredness, physical stress or injury, or other related complications. The Prehab program is in addition to usual care and typically patients with inguinal hernia do not participate in physical exercise prior to undertaking surgery. However, the program comes with several potential benefits that will be discussed.

Specific physical complications could arise before the operation is performed or after surgery. Prior to surgery, the patient may experience more pain in the hernia site as well as normal pain associated with exercise, they may notice worsening of the size of the hernia, and there is a very small chance of hernia incarceration (i.e. herniated tissue becomes trapped and cannot easily be moved back into place – typically occurs in less than 1% (0.18% to 0.79%) of hernia cases). Increased symptoms or size of the hernia will be monitored, and exercises will be modified if the symptoms become intolerable to the participant. Hernia incarceration is an acute painful episode that will require a visit with the surgeon.

In the post-operative phase, patients may experience postoperative pain, opening of the superficial wound from potential hazards associated with exercising (i.e. falling, etc.). However, studies on the force required to cause hernia rupture indicate the force is quite high and beyond those generated

during the exercises prescribed. If a participant experiences pain associated with our exercise intervention, we will modify or stop the aggravating exercises after consultation with the surgeon.

Exercise prior to surgery requires mental concentration and participants may feel psychologically or emotionally stressed or fatigue. Since they are in a research study, there is always some risk of social risk through loss of privacy.

4.0 * **Describe how you will manage and minimize risks and discomforts, as well as mitigate harm:**
The exercise programs will be monitored by experienced physical therapists and kinesiologists who will track patient progress. Since the exercise program is overall low intensity exercise, we do not anticipate many injuries or adverse events. However, if patients begin experiencing problems, the therapists will advise on alterations to make the exercises less intensive or recommend that the program be stopped. The therapists will also be available should more serious adverse events occur such as injury or other related complication. If this occurs, the therapists will advise on self-management strategies for the injury or initiate follow-up with the surgeon.

5.0 **Is there a possibility that your research procedures will lead to unexpected findings, adverse reactions, or similar results that may require follow-up** *(i.e. individuals disclose that they are upset or distressed during an interview/questionnaire, unanticipated findings on MRI, etc.)***?**

● **Yes** ○ No

Describe the arrangements or referral the researcher will make. Explain if no arrangements have been made.
As mentioned above, the participants will be monitored by experienced physical therapists and kinesiologists that can provide advice or take immediate action. The general surgeon on our team who are helping to recruit patients will also be available to consult on any serious adverse reactions.

6.0 **If you are using any tests in this study diagnostically, indicate the member(s) of the study team who will administer the measures/instruments:**

Test Name	Test Administrator	Organization	Administrator's Qualification

There are no items to display

7.0 **If any research related procedures/tests could be interpreted diagnostically, will these be reported back to the participants and if so, how and by whom?**
Not applicable as the measures we are using track patient progress only. They are not interpreted diagnostically.

If the surgeon believes an ultrasound imaging scan is needed to detect re-of the hernia, this will be done in the context of usual care and results will be reported to the patients.

3.2 Benefits Analysis

1.0 * **Describe any potential benefits of the proposed research to the participants. If there are no benefits, state this explicitly:**
The patient centred evidence-based perioperative program that participants will be undertaking has the potential to: 1) improve post-surgical pain; 2) increase post-surgical functional and patient oriented outcomes; 3) decrease post-surgical opioid use; and 4) decrease short- and long-term healthcare costs by reducing readmissions, and post-operative health utilization.

2.0 * **Describe the scientific and/or scholarly benefits of the proposed research:**
This study is novel and timely given the current healthcare focus on improving prevention strategies, and improving access to, delivery and outcomes of surgical interventions. This will be the first perioperative program specifically developed for patients undergoing surgery for inguinal hernia. The obvious potential benefits for patients undergoing inguinal hernia repair is the development of a program that has the potential to improve post-surgical outcomes, increase quality of life, while addressing healthcare and individual costs, minimizing the impact of the condition on the individual and society.

3.0 **If this research involves risk to participants explain how the benefits outweigh the risks.**
While the perioperative program comes with some inherent risks due to participation in exercise, this exercise also comes will several potential benefits for participants. Since the risk of adverse events are quite small, we believe the potential benefits outweigh the risks.

4.1 Participant Information

1.0 * **Will you be recruiting human participants** *(i.e. enrolling people into the study, sending people online surveys to complete)***?**

● **Yes** ○ No

1.1 Will participants be recruited or their data be collected from Alberta Health Services or Covenant Health or data custodian as defined in the Alberta Health Information Act?

● **Yes** ○ No

1.2 Would you like to include information about this study on the Be The Cure searchable database?

○ Yes ● **No**

4.2 Additional Participant Information

1.0 **Describe the participants that will be included in this study. Outline ALL participants** *(i.e. if you are enrolling healthy controls as well):*

We will recruit 30 participants with inguinal hernia who are scheduled for surgery by a general surgeon in Fort Saskatchewan (Dr. Omar Farooq). *We will recruit 30 participants in total and randomize 15 participants to each of the intervention and control groups.*

2.0 * **Describe and justify the inclusion criteria for participants** *(e.g. age range, health status, gender, etc.):*

Inclusion criteria include:

1) scheduled to undergo first-time inguinal hernia repair surgery after a physical examination identified signs and symptoms consistent with inguinal hernia (direct or indirect hernia);

2) willingness to participate in a 6-week targeted exercise program;

3) 18+ years of age;

4) no medical contraindications to participation in exercise. *Contra-indications that will be screened for include associated medical conditions that preclude exercise. This includes uncontrolled medical conditions including diabetes, hypertension, vertigo, congestive heart failure, chronic obstructive pulmonary disease, intra-abdominal ascites, or pre-existing malnutrition.*

and

5) employed full-time and required to lift at least 10kg at work.

3.0 **Describe and justify the exclusion criteria for participants:**

Exclusion criteria include: 1) previous inguinal hernia;

2) Body Mass Index >35 since morbidly obese patients experience more surgical complications

3) use of narcotics

4) bone and joint conditions of the spine or extremities

5) history of other abdominal surgeries

4.0 **Participants**

4.1 How many participants do you hope to recruit *(including controls, if applicable?)*
30
4.2 Of these, how many are controls, if applicable?
0

4.3 If this is a multi-site study, how many participants do you anticipate will be enrolled in the entire study?
30

5.0 Justification for sample size:
Since this is a pilot study evaluating feasibility of our intervention and evaluation protocols, we will aim to enrol 30 patients. We will randomize 15 participants to each group. Thirty participants has been deemed acceptable to yield confidence intervals whose lower limits can help define the range of plausible values for a subsequent power analysis. This will also provide adequate estimates of enrolment, follow-up rates, information on timing, and variance in our measurement protocol to inform a subsequent randomized controlled trial of the intervention.

4.3 Recruitment of Participants (Health)

1.0 Recruitment

* **1.1 How you will identify potential participants? Please be specific.** *(i.e. Will you be screening clinical lists, accessing electronic health records (e-clinician), asking staff from a particular area to let you know when a patient meets criteria, will you be sitting in the emergency department waiting room, etc?*)
Recruitment will take place at the general surgical practice of Dr. Farooq at the Fort Saskatchewan Community Hospital. Patients on the wait list for inguinal hernia repair surgery will be contacted by Dr. Farooq's administrative support staff. They will ask the participant if they are interested in participating in this study. If so, their contact information will be taken and provided to the study team who will then contact the individual to provide further study information and obtain informed consent.

1.2 If you are using patient/clinical records to identify potential participants for research purposes, will someone from the data custodian/clinical care team seek prior consent of the participant to allow the researcher to look at their records?
● **Yes** ○ No

1.3 Once you have identified a list of potentially eligible participants, indicate how the potential participants' names will be passed on to the researchers AND how will the potential participants be approached about the research.
The names and contact details of potentially eligible participants will be provided to the investigative team by the surgeon's research coordinators using a 'Consent to Contact' form that will be completed either at time of their examination by the surgeon or after they are on the wait list and contacted by the administrative support staff. If contacted over the phone, the consent to contact form will be emailed for signing. The investigators will

then arrange a meeting (telephone or Zoom videoconference) with the potential participant to explain the study and provide an information sheet. Final consent to participate will be done over REDcap.

1.4 Outline any other means by which participants could be identified*(e.g. response to advertising such as flyers, posters, ads in newspapers, websites, email, list serves, physicial or community organization referrals):*
Not applicable since we require patients who have been scheduled for inguinal repair surgery.

2.0 Pre-Existing Relationships

2.1 Will potential participants be recruited through pre-existing relationships with researchers *(e.g. Will an instructor recruit students from his classes, or a physician recruit patients from her practice? Other examples may be employees, acquaintances, own children or family members, etc)***?**

● **Yes** ○ No

2.2 If YES, identify the relationship between the researchers and participants that could compromise the freedom to decline *(e.g. clinician/patient, professor/student):*
If it is acceptable to the ethics board, Dr, Farooq will recruit directly. However, we will have a research assistant make first contact with patients on Dr. Farooq's surgical wait list so that patients feel no obligation or coercion to participate in the study.

2.3 How will you ensure that there is no undue pressure on the potential participants to agree to the study?
Initial contact will be made by an administrative staff member to determine if the potential participants are interested.

3.0 Will your study involve any of the following *(select all that apply)***?**
None of the above

4.5 Informed Consent Determination

1.0 Describe who will provide informed consent for this study*(i.e. the participant, parent of child participant, substitute decision maker, no one will give consent – requesting a waiver)*
Each participant will provide informed consent for this study. Since the first session is in-person, we will complete the consent process and documentation in person. Although the participants may also be asked to complete the study demographic form and questionnaires via REDCap prior to that session (consent implied through completion).

1.1 Waiver of Consent Requested
If you are asking for a waiver of participant consent, please justify the waiver or alteration and explain how the study meets all of the criteria

for the waiver. Refer to Article 3.7 of TCPS2 and provide justification for requesting a Waiver of Consent for ALL criteria (a-e)
Not applicable.

1.2 Waiver of Consent in Individual Medical Emergency
If you are asking for a waiver or alteration of participant consent in individual medical emergencies, please justify the waiver or alteration and explain how the study meets ALL of the criteria outlined in Article 3.8 of TCPS2 (a-f).
Not applicable.

2.0 **How will consent be obtained/documented? Select all that apply**
Signed consent form

If you are not using a signed consent form, explain how the study information will be provided to the participant and how consent will be obtained/documented. Provide details for EACH of the options selected above:

3.0 **Will every participant have the capacity to give fully informed consent on his/her own behalf?**
● **Yes** ○ No

4.0 **What assistance will be provided to participants or those consenting on their behalf, who may require additional assistance?** *(e.g. non-English speakers, visually impaired, etc.)*
The study team will meet with the participants to provide any assistance needed. However, due to the nature of this study any participants experiencing difficulty with the consent process will not be enrolled.

5.0 * **If at any time a PARTICIPANT wishes to withdraw from the study or from certain parts of the study, describe when and how this can be done.**
Participants can withdraw at any time during the study. If requested, all electronic data will be deleted for those withdrawing.

6.0 **Describe the circumstances and limitations of DATA withdrawal from the study, including the last point at which participant DATA can be withdrawn** *(i.e. 2 weeks after transcription of interview notes)*
Participants can withdraw from the study up to the point where analysis has been completed and results presented.

7.0 **Will this study involve any group(s) where non-participants are present? For example, classroom research might involve groups which include participants and non-participants.**
○ Yes ● **No**

5.1 Data Collection

1.0 * **Will the researcher or study team be able to identify any of the participants at <u>any stage</u> of the study?**

● **Yes** ○ No

2.0 **Primary/raw data collected will be** *(check all that apply)*:
All personal identifying information removed (anonymized)

3.0 **If this study involves secondary use of data, list all original sources:**
Not applicable.

4.0 **In research where total anonymity and confidentiality is sought but cannot be guaranteed** *(eg. where participants talk in a group)* **how will confidentiality be achieved?**
Not applicable.

5.2 Data Identifiers

1.0 * **Personal Identifiers:** will you be collecting - at any time during the study, including recruitment - any of the following *(check all that apply)*:
Surname and First Name
Address
Full Postal Code
Telephone Number
Email Address
Age at time of data collection

2.0 **Will you be collecting - at any time of the study, including recruitment of participants - any of the following** *(check all that apply)*:
There are no items to display

3.0 * **If you are collecting any of the above, provide a comprehensive rationale to explain why it is necessary to collect this information:**
Name and contact information will be used to recruit participants.
Age at time of data collection is being collected to describe our sample.
Address and postal code will be used to contact patients at follow-up.

4.0 **If identifying information will be removed at some point, when and how will this be done?**
Personal identifying information will not be removed, since we will need it to contact participants at follow-up.

5.0 * **Specify what identifiable information will be RETAINED once data collection is complete, and explain why retention is necessary. Include the retention of master lists that link participant identifiers with de-identified data:**
All patient names and contact information will be retrained in case we need to follow-up with patients about the study.

6.0 **If applicable, describe your plans to link the data in this study with data associated with other studies (e.g within a data repository) or with data belonging to another organization:**
Not applicable.

5.3 Data Confidentiality and Privacy

1.0 * **How will confidentiality of the data be maintained? Describe how the identity of participants will be protected both during and after research.**
All investigators and graduate students working on this project will be trained in research ethics (CORE certification) and confidentiality issues. All data will be stored on password-protected and encrypted secure computers at the University of Alberta and McMaster University campuses.
During presentations, we present group-level data and avoid providing individual identifiers.

2.0 **How will the principal investigator ensure that all study personnel are aware of their responsibilities concerning participants' privacy and the confidentiality of their information?**
All investigators and graduate students working on this project will be trained in research ethics (CORE certification) and confidentiality issues.

3.0 **External Data Access**

* **3.1 Will identifiable data be transferred or made available to persons or agencies outside the research team?**
○ Yes ● No

5.4 Data Storage, Retention, and Disposal

1.0 * **Describe how research data will be stored, e.g. digital files, hard copies, audio recordings, other. Specify the physical location and how it will be secured to protect confidentiality and privacy.** *(For example,*

study documents must be kept in a locked filing cabinet and computer files are encrypted, etc. Write N/A if not applicable to your research)
All electronic data will be saved in a password protected, secure University of Alberta computer and network within a password protected Excel file. All physical documents will be stored in the Rehabilitation Research Centre (Room 3-62 Corbett Hall) at the University of Alberta. Only the Co-PIs and their graduate students will have access to study data.

2.0 * **University policy requires that you keep your data for a minimum of 5 years following completion of the study but there is no limit on data retention. Specify any plans for future use of the data. If the data will become part of a data repository or if this study involves the creation of a research database or registry for future research use, please provide details.** *(Write N/A if not applicable to your research)*
We have no plans for future use of the data, but will keep all records and data for a period of 5 years at least.

3.0 **If you plan to destroy your data, describe when and how this will be done? Indicate your plans for the destruction of the identifiers at the earliest opportunity consistent with the conduct of the research and/or clinical needs:**
We have no plans for destroying our data.

Documentation

Add documents in this section according to the headers. Use Item 11.0 "Other Documents" for any material not specifically mentioned below.

Sample templates are available by clicking HERE.

1.0 **Recruitment Materials:**

Document Name	Version	Date	Description
Consent to Contact form V2	0.03	2021-03-02 4:43 PM	
CLEAN Consent to Contact form V2	0.02	2021-03-03 9:01 AM	

2.0 **Letter of Initial Contact:**

Document Name	Version	Date	Description

There are no items to display

3.0 **Informed Consent / Information Document(s):**

3.1 What is the reading level of the Informed Consent Form(s):
9

3.2 Informed Consent Form(s)/Information Document(s):

Document Name	Version	Date	Description
Letter of Information and Consent V2	0.05	2021-03-02 4:43 PM	
CLEAN Letter of Information and Consent V2	0.01	2021-03-03 7:47 AM	

4.0 Assent Forms:

Document Name	Version	Date	Description

There are no items to display

5.0 Questionnaires, Cover Letters, Surveys, Tests, Interview Scripts, etc.:

Document Name	Version	Date	Description
Assessment booklet V4.docx	0.03	2021-03-02 4:44 PM	
Feedback Survey	0.01	2021-03-02 4:44 PM	
Functional Testing Form	0.01	2021-03-02 4:45 PM	
Adherence and Adverse Events Form	0.01	2021-03-02 4:46 PM	
CLEAN Assessment booklet V4.docx	0.01	2021-03-03 7:48 AM	

6.0 Protocol/Research Proposal:

Document Name	Version	Date	Description
Full proposal	0.04	2021-01-13 12:57 PM	
Data Collection Timeline and Protocol	0.01	2021-03-02 4:45 PM	
Exercise Protocol	0.01	2021-03-02 4:46 PM	

7.0 Investigator Brochures/Product Monographs:

Document Name	Version	Date	Description

There are no items to display

8.0 Health Canada No Objection Letter *(NOL)*:

Document Name	Version	Date	Description

There are no items to display

9.0 Confidentiality Agreement:

Document Name	Version	Date	Description

There are no items to display

10.0 Conflict of Interest:

Document Name	Version	Date	Description

There are no items to display

11.0 Other Documents:
For example, Study Budget, Course Outline, or other documents not mentioned above

	Document Name	Version	Date	Description
	Appendix with Feasibility Criteria	0.02	2021-01-13 12:25 PM	

Final Page

You have completed your ethics application! Click "Continue" to go to your workspace.

This action will NOT SUBMIT the application for review.

Only the Study Investigator can submit an application to the REB by selecting the "SUBMIT STUDY" under My Activities after you click CONTINUE.
